# Ectopic Hidradenoma Papilliferum

**DOI:** 10.1155/2010/709371

**Published:** 2010-12-12

**Authors:** Aristóteles David Neiva Rosmaninho, Maria Teresa Duarte Pinto de Almeida, Vírgilio Costa, Maria Madalena Vasconcelos Sanches, Carlos Lopes, Maria Manuela Selores Gomes Meirinhos

**Affiliations:** ^1^Departamento de Dermatologia, Centro Hospitalar do Porto, HSA, Edifício das Consultas Externas, Ex-CICAP, Rua D. Manuel II, 4099-001 Porto, Portugal; ^2^Departamento de Anatomia Patológica, Centro Hospitalar do Porto, HSA, Porto, 4099-001 Porto, Portugal

## Abstract

Hidradenoma papilliferum is a rare tumor that occurs almost exclusively in females on the anogenital area. Rare cases of ectopic (nongenital) hidradenoma papilliferum have been described. The lesions usually present as an asymptomatic slow-growing, red, firm, mobile, well-delimitated nodule that grows for a long time before resection. We describe a case of an 26-year-old man that presented with an enlarging nodule on his right eyelid. The histological findings revealed a hidradenoma papilliferum. So far, among the very few reports of ectopic hidradenoma papilliferum, only a very small number were localized to the eyelid.

## 1. Introduction

Hidradenoma papilliferum is a slow-growing benign adnexal tumor with apocrine differentiation, and for some authors it can be considered an analog of intraductal papilloma of the breast [1]. The tumor primarily affects vulvar, perineal and perianal skin of middle-aged women with rare cases being reported in other skin localizations (ectopic hidradenoma papilliferum) [2, 3]. To our knowledge, only a perianal, hidradenoma papilliferum has been described in a man [4]. The clinical presentation as well as the pathologic features, treatment, and prognosis are similar in both forms. It usually presents as a slow-growing, solitary, asymptomatic skin colored or red nodule less than 1 cm in diameter [5]. However, a giant ectopic hidradenoma papilliferum on the scalp has been recently described [6]. The most common site of ectopic hidradenoma papilliferum is the head and neck. The diagnosis can be made only by histopathological examination since they clinically mimic other cutaneous neoplasms.

## 2. Clinical History

**Figure 1:**
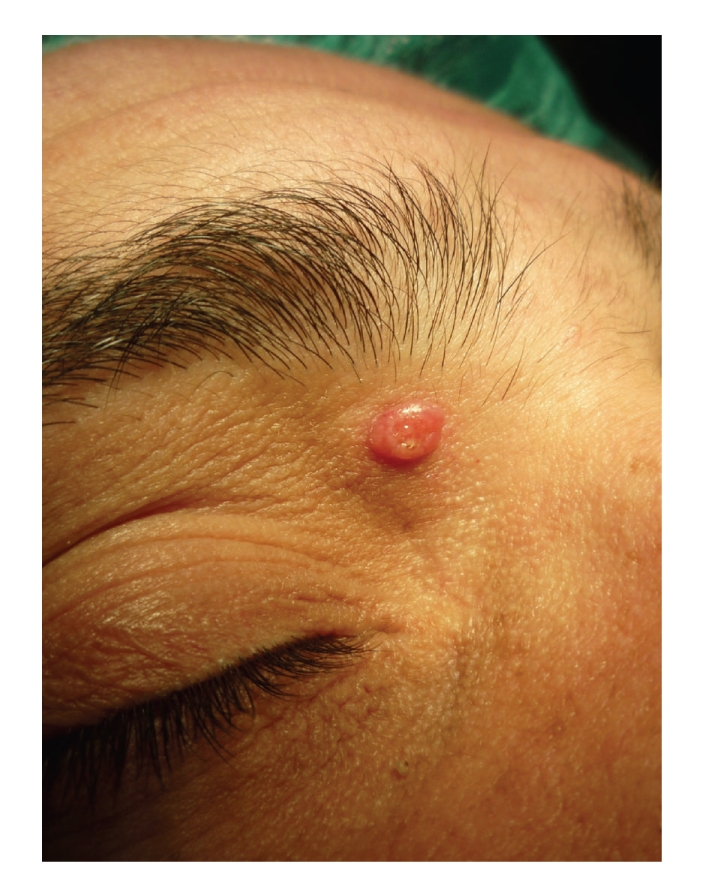
Clinical features of the skin lesion on the upper eyelid.

**Figure 2:**
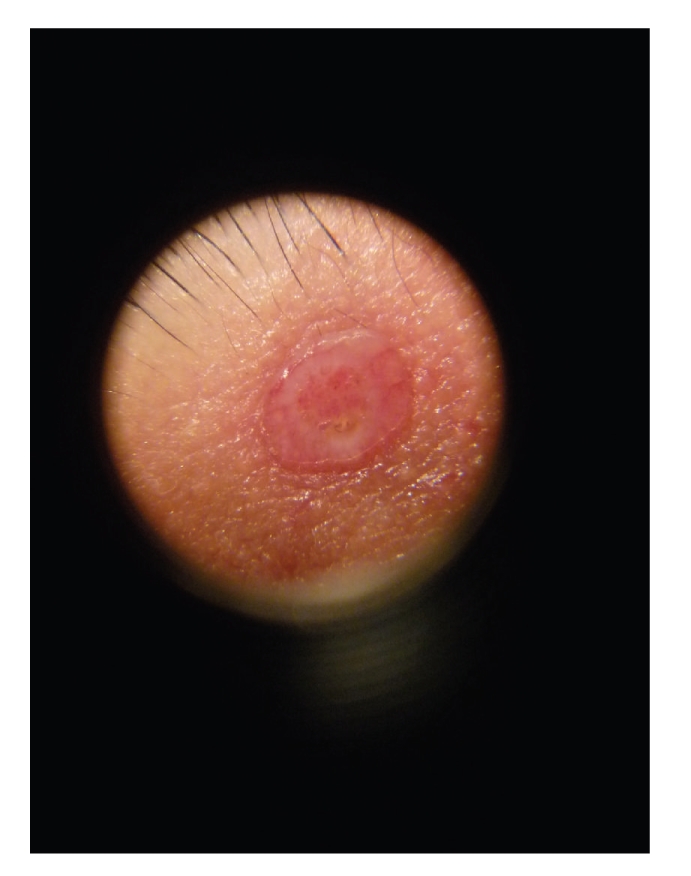
Dermoscopy findings.

A 26-year-old white man presented in our department with a 2-year history of an asymptomatic enlarging lesion of the upper eyelid. No previous lesion, namely nevus sebaceous was documented. His personal and familiar medical history was unremarkable. On physical examination a well-circumscribe, firm, umbilicated, reddish, molluscum contagiosum-like nodule approximately 1 cm in diameter localized to the upper right eyelid was observed (Figure 1). Dermoscopy showed several telangiectasias in the surface of the nodule and a central umbilication (Figure 2). A basal cell carcinoma or an adnexal skin tumor was suspected. Surgical excision of the lesion was performed and the specimen was sent for microscopic examination. 

**Figure 3:**
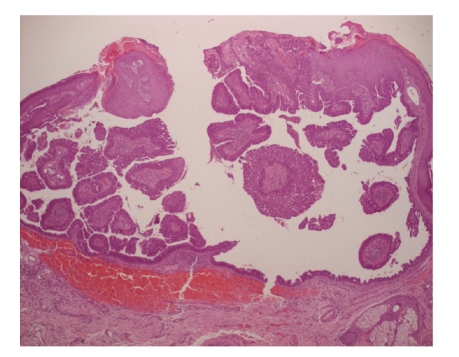
Hematoxylin-eosin stain (4x) showed a dermal nodule with many glandular structures and papillary folds.

**Figure 4:**
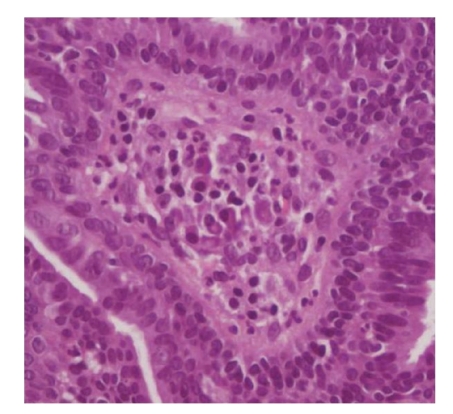
Hematoxylin-eosin stain (40x) showed the presence of focal areas infiltrated by plasma cells and lymphocytes in the stroma.

**Figure 5:**
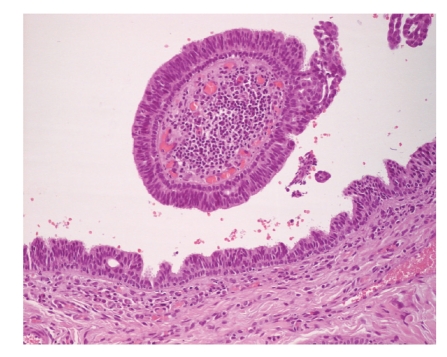
Hematoxylin-eosin stain (20x) showed the tumor epithelium to be composed by an outer cuboidal cell layer and an inner columnar cell layer.

Microscopic examination showed a well-circumscribed dermal nodule characterized by a large cystic space containing eosinophilic debris and papillary folds. The lesion seemed to be connected with the epidermis and a central ulceration was observed. Papillae and micropapillae projected from the cyst wall into its cavity (Figure 3). The papillae had a broad fibrous core with aggregates of lymphocytes and few plasma cells (Figure 4). A basal layer composed by small cuboidal cells and a luminal layer composed by larger columnar cells was observed (Figure 5). These microscopic features were consistent with the diagnosis of hidradenoma papilliferum.

## 3. Discussion

Hidradenoma papilliferum is originated in the apocrine glands, which are mainly concentrated in the anogenital region, axillae, and periumbilical areas. The tumor occurs mainly in those areas with ectopic localization being rarely reported [2]. The distribution of the ectopic forms corresponds to the areas containing heterotrophic and modified apocrine glands. According to a medline search, only 20 reports of ectopic hidradenoma papilliferum have been described in the English language and only 3 were localized to the eyelid [7–9]. Independently of being typical or ectopic the tumor occurs mostly in white women. However, in contrast to anogenital hidradenoma papilliferum, nearly one-half of the patients with ectopic hidradenoma papilliferum are men [2]. The head and neck are the most frequent localization for the ectopic presentation, mainly the eyelid and external ear, where modified apocrine glands (Moll and ceruminous glands) are found normally [2, 7]. Sometimes ectopic apocrine tumors are also found in the scalp within lesions such as the nevus sebaceous of Jadassohn. There are 4 reported cases of hidradenoma papilliferum on the head and neck region in males [7]. Other ectopic localizations included arm, thigh, back, and nipple [2, 7, 10]. The age range reported is between 8 to 78 years [7]. The clinical presentation of both forms is similar to most lesions, being asymptomatic and growing for a long time before excision. Pain, pruritus or, ulceration can occur. Like other adnexal skin tumors they clinically mimic other neoplasms such as basal cell carcinoma (as in our case report) and spinocellular carcinoma. Thus, histological examination is required for the correct diagnosis. Histologically the tumor is characterized by a cystic space containing eosinophilic material and papillary folds projected from the cyst wall. Tumor epithelium is composed by a basal layer of cuboidal cells and a luminal layer of larger columnar cells showing decapitation secretion [11]. The epidermis may be normal, acanthotic, or ulcerated and may sometimes show continuity with the overlying epithelium [12, 13]. In some cases the tumor displays a histopathology similar to syringocystadenoma papilliferum since they are closely related tumors which originate from apocrine glands [14]. Aggregates of lymphocytes and plasma cells have been described in the stroma of ectopic lesions [2]. Some authors considered the presence of focal areas infiltrated by plasma cells and lymphocytes as a sign of a mixed differentiation between hidradenoma papilliferum and syringocystadenoma papilliferum [7]. A report of an ectopic hidradenoma papilliferum with sebaceous differentiation has been documented [8]. Other histopathological differential diagnosis includes tubular apocrine adenoma and clear cell (apocrine) adenoma. The prognosis is good with local excision being the treatment of choice. Recurrence of the lesions is attributed to incomplete excision of the primary tumor and there is no report of recurrence for the ectopic form [2]. Malignant transformation in anogenital hidradenoma papilliferum has been documented (intraductal carcinoma resembling apocrine carcinoma and invasive adenosquamous carcinoma) but not in the ectopic presentation [2, 15, 16]. It is speculated that HPV may play a role in inducing malignancy, but the association still needs to be proved [17]. We presented a new case of ectopic hidradenoma papilliferum with features of a mixed differentiation arising in the eyelid.
